# aMMP-8 POCT for Periodontal Disease: An Indicator of Poor Oral Health

**DOI:** 10.1177/10732748231214874

**Published:** 2023-11-15

**Authors:** Shipra Gupta, Ismo T. Räisänen, Hanna Seppänen, Jaana Hagström, Andreas Grigoriadis, Kethe Hermunen, Dimitra Sakellari, Caj Haglund, Timo Sorsa

**Affiliations:** 1 Oral Health Sciences Centre, 29751 Post Graduate Institute of Medical Education & Research, Chandigarh, India; 2 Department of Oral and Maxillofacial Diseases, 3835 University of Helsinkiand Helsinki University Central Hospital, Helsinki, Finland; 3 Research Program Unit, Translational Cancer Medicine, 3835 University of Helsinki, Helsinki, Finland; 4 Department of Surgery, 3835 University of Helsinkiand Helsinki University Hospital, Helsinki, Finland; 5 iCAN Digital Precision Cancer Medicine Flagship, 3835 University of Helsinki, Helsinki, Finland; 6 Department of Pathology, Haartman Institute and HUSLab, 3835 University of Helsinkiand Helsinki University Hospital, Helsinki, Finland; 7 Department of Oral Pathology and Radiology, University of Turku, Turku, Finland; 8 Department of Preventive Dentistry, Periodontology and Implant Biology, Dental School, 37782 Aristotle University of Thessaloniki, Thessaloniki, Greece; 9 424 General Army Hospital, Thessaloniki, Greece; 10 Division of Periodontology, Department of Oral Diseases, Karolinska Institutet, Sweden

**Keywords:** pancreatic cancer, oral health, risk factors, oral hygiene, matrix metalloproteinases

## Letter to the Editor

In a recent editorial by Dr Gaëtan Romain Joliat entitled ‘Latest Advances and Future Challenges in Pancreatic Surgery’, the authors stress on how development and improvement in the fields of chemotherapy, targeted therapy and immunotherapy will benefit patients with pancreatic cancers.^1^ We as dentists and surgeons would like to bring to the notice of our medical colleagues periodontal disease as yet another co-risk factor of pancreatic cancer.

Yu et al. (2022) also confirm the association between poor oral health and pancreatic cancer risk. They found a statistically significant association between periodontitis and an increased risk of pancreatic cancer in patients under the age of 50 (*P* < .001) as well as in patients between 50 and 70 (multivariable-adjusted HR = 1.20, 95% confidence interval [CI] 1.11-1.29).^2^ Periodontal disease, tooth loss and root canal infections showed a positive association with an increased risk of developing pancreatic cancer. Previously, Heikkilä et al. (2018), in their register-based cohort study of 68 273 adults, reported a higher pancreatic cancer mortality among individuals with periodontitis (crude rate ratios: RR 1.69, 95% CI 1.04-2.76).^3^ An even stronger association was noted after adjustments (RR 2.32, 95% CI 1.31-3.98). Fan et al. (2018) in their population-based nested case-control study have provided supportive evidence towards the role of oral periodontopathogenic microbiota in the aetiology of pancreatic cancer.^4^

Evidence shows that periodontopathogens like *Porphyromonas gingivalis*, *Treponema denticola* (Td), *Fusobacterium nucleatum* and *Tannerella forsythia* have a more potent role in the causation of cancers of the digestive tract than previously thought. They are able to evade the immune mechanisms and colonise the gastrointestinal tract, disrupting the epithelial integrity. Of these, Td is a notoriously invasive motile anaerobe with an equally harmful surface-bound chymotrypsin-like proteinase (CTLP, also known as dentilisin) virulence factor.^5^ The possible mechanisms by which it can promote carcinogenesis include (a) hydrolysis of bioactive peptides; (b) inhibition of apoptosis; (c) promotion of cellular invasion; (d) degradation of multiple host proteins; and (e) modulate immunity and inflammation.^6^

Additionally, research has found that certain potent periodontopathobionts are implicated in the tumorigenesis and progression of cancers affecting not only the oral cavity but also oesophageal, breast and gall bladder carcinomas. According to Nwizu et al. (2020), periodontal disease raises the general cancer risk by 14%.^7^ Females with periodontal disease were reported to be 3 times more likely to develop oesophageal cancer. Hence, periodontal disease could be a significant risk factor for cancer, especially in mature women.

Patients with pre-existing gum disease are currently likely to have an increased risk for pancreatic cancer. As periodontal disease is a modifiable risk factor, it is of uppermost importance that regular and valid screening measures are employed for its early detection.

To further add to the knowledge, dentilisin acts against various immunomodulatory proteins critical for the regulation of tumour microenvironment and inflammation, thereby further contributing to tumour progression. It also has the ability to modulate immunomodulatory proteins, including matrix metalloproteinases (MMPs) and tissue inhibitors of MMPs (TIMPs), both of which have been implicated in regulation of tumour microenvironment and metastasis of gastrointestinal cancers.^6,8,9^

MMPs are derived from host PMNs, endothelial, epithelial and smooth muscle cells in latent pro-MMP form. Of all MMPs, particularly activity of MMP-8 (collagenase) is elevated in patients suffering from gum disease as it causes destruction and digestion of type I collagen which is present in periodontal connective tissue.^10^

**Figure 1.**
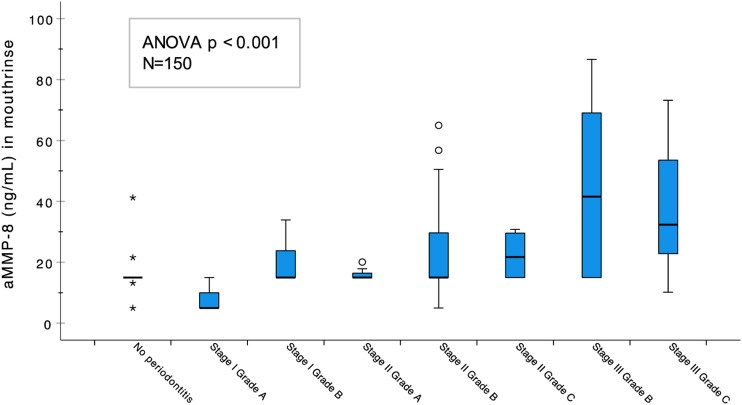
Significant association between aMMP-8 levels in mouthrinse and the severity of stage and grade of periodontal disease diagnosis (ANOVA *P* < .001). The aMMP-8 levels measured by aMMP-8 POCT increased according to the severity of disease status among 150 patients that have been previously described in Sorsa et al. (2021).

Cleavage of latent MMP-8 by periodontal microbes and their virulence factors like Td-dentilisin leads to release of active MMP-8 molecules and also smaller MMP-8 fragments which are detectable by active MMP-8 point-of-care test (aMMP-8 POCT) kits readily available in the market.^5,11-13^ Elevated aMMP-8 levels in mouthrinse and patients’ periodontal disease can be conveniently detected by aMMP-8 POCT (Figure 1). These are lateral flow immunoassay-based kits utilising detection of aMMP-8 in oral fluids like gingival crevicular fluid and mouthrinse.^12,13^ The sensitivity of the aMMP-8 POCT is 75%-85% and specificity is 80%-90%.^14^ There is enough evidence in literature to indicate that they are valuable tools for screening periodontal status of an individual in a predictable manner. These kits can hence be utilised to screen patients conveniently and in a timely manner.

The authors recommend utilising non-invasive biomarkers like aMMP-8 for early detection of periodontal disease since it is a risk factor for carcinomas.
